# A Facile Synthesis of RGO-Ag_2_MoO_4_ Nanocomposites for Efficient Lead Removal from Aqueous Solution

**DOI:** 10.3390/molecules29215152

**Published:** 2024-10-31

**Authors:** Mohd Shoeb, Fouzia Mashkoor, Mohmmad Naved Khan, Changyoon Jeong

**Affiliations:** School of Mechanical Engineering, Yeungnam University, Gyeongsan 38541, Republic of Korea; mshoeb@yu.ac.kr (M.S.); fmashkoor@yu.ac.kr (F.M.); navedkhan@yu.ac.kr (M.N.K.)

**Keywords:** reduced graphene oxide, lead, molybdenum oxide, silver, wastewater, adsorption

## Abstract

Efficiently treating wastewater, particularly the elimination of heavy metal ions from water systems, continues to be one of the most pressing and complex challenges in modern environmental management. In this work, reduced graphene oxide coupled silver molybdate binary nanocomposites (RGO-Ag_2_MoO_4_ NCs) have been prepared via hydrothermal method. The crystalline nature and surface properties of the developed RGO-Ag_2_MoO_4_ NCs were proved by XRD, FTIR, SEM, and EDS techniques. Adsorption experiments demonstrated that the nanocomposites (NCs) effectively removed Pb(II) ions within 120 min, achieving a maximum removal efficiency ranging from 94.96% to 86.37% for Pb(II) concentrations between 20 and 100 mg/L at pH 6. Kinetic studies showed that the adsorption process followed a pseudo-second order model. Isotherm analysis presented that the Langmuir model provided the greatest fit for the equilibrium data, with a monolayer adsorption capacity of 128.94 mg/g. Thermodynamic analysis revealed that the adsorption process was spontaneous and endothermic. The results of this study highlight RGO-Ag_2_MoO_4_ NCs as a highly promising and eco-friendly material for the effective elimination of Pb(II) ions from wastewater. Their strong adsorption capacity, coupled with sustainable properties, makes them an efficient solution for addressing lead contamination, offering significant potential for practical applications in water treatment systems.

## 1. Introduction

Water is a vital resource for sustaining life and supporting economic and social development. However, the rapid global population growth, along with increasing demands from agriculture, industry, and urbanization, has placed immense strain on water resources [1]. This strain is further exacerbated by the contamination of water supplies with various pollutants, particularly heavy metals released from industrial activities such as mining, metal refining, and pesticide use [2]. Among these pollutants, lead is a major concern owing to its widespread presence in water sources and serious health risks. Lead contamination in water is a global issue, largely driven by human activities like mining, the improper disposal of industrial waste, and the use of lead-based materials [3]. Lead typically exists in its ionic form, Pb(II), which is highly toxic and difficult to remove from water. Prolonged exposure to lead-contaminated water can result in serious health issues, including neurological disorders, behavioral disorders, weakness, developmental delays in children, and cardiovascular and constipation problems [4]. This makes the mitigation of lead pollution and the improvement of drinking water quality a critical and urgent priority for public health.

Various techniques are utilized to eliminate harmful substances from polluted water, such as electrodialysis, chemical precipitation, photocatalysis, and membrane filtration [5,6]. Despite their widespread use, these traditional methods often come with notable limitations, including high energy requirements, incomplete pollutant removal, expensive machinery, and the production of sludge or solid waste that necessitates additional disposal efforts. Furthermore, they may struggle to handle large quantities of wastewater, which hampers their applicability on a larger scale. Among the different methods, adsorption has gained recognition as a highly efficient and environmentally friendly solution for extracting heavy metals like lead from water. This approach is not only economical but also features a straightforward design and operational flexibility, allowing for the regeneration of the adsorbents used [7]. The efficiency of adsorption largely depends on the characteristics of the adsorbent material, which play a crucial role in attaining optimal performance. Recent studies are concentrating on creating advanced adsorbents with enhanced capacities for contaminants uptake and improved regeneration capabilities for larger-scale applications. Innovations such as hybrid adsorbents, which integrate the benefits of various materials, along with functionalized nanomaterials, are being explored to refine water treatment methods. These developments are critical for meeting the increasing need for effective, scalable, and sustainable water purification solutions, especially for communities affected by organic and inorganic contamination [8].

Metal oxides have been widely used in environmental remediation due to their unique physicochemical properties, such as high surface area, chemical stability, and reactivity towards various pollutants [9]. Hybridizing metal oxides with other materials has shown to enhance these properties even further, leading to more effective solutions for pollutant removal. Among the various metal oxides, molybdenum oxide (MoO_4_) has gained attention for its high potential in the adsorptive removal of contaminants like lead and other heavy metal ions. Molybdenum-based nanomaterials are valued for their unique optical, electronic, mechanical, and catalytic characteristics, which enhance their effectiveness in pollutant removal [10]. Specifically, MoO_4_ exhibits advantageous features such as uniform nanoscale size, resistance to acidic conditions, thermal stability, and low cytotoxicity, all of which contribute to its high performance in water treatment applications. The oxygen-rich structure of MoO_4_ provides numerous active sites, enabling strong interactions with heavy metal ions [11]. However, the direct use of MoO_4_ is limited by its high solubility in water, which can lead to leaching and secondary pollution. To mitigate this issue, MoO_4_ is often incorporated into composite materials or hybridized with stable supports, such as polymers, other metal oxides, or carbon-based materials. This hybridization improves its adsorption capacity, selectivity, and stability, reducing environmental risks and enhancing the material’s overall performance [12,13,14]. Enhancing MoO_4_ with the addition of silver (Ag) significantly boosts its ability to adsorb heavy metal ions, particularly lead. Silver atoms create extra active sites on the surface of the MoO_4_, which improves its capacity to capture and bind lead ions more effectively. This modification not only increases the overall adsorption capability of MoO_4_ but also improves its structural stability and reactivity, making the material more resilient and efficient under challenging environmental conditions. Consequently, the combination of MoO_4_ and silver forms a more powerful and durable adsorbent for removing heavy metal contaminants from water, making it highly effective for environmental remediation.

Incorporating reduced graphene oxide (RGO) into the MoO_4_-Ag composite further enhances the material’s properties, providing several additional benefits. RGO is well known for its high surface area and superior electrical conductivity, which facilitate efficient electron transfer during the adsorption process [15]. This improvement leads to faster adsorption rates, allowing for the quick removal of lead from water. Furthermore, the presence of oxygen-containing functional groups in RGO enhances its interactions with HMIs, thereby increasing the composite’s adaptability in addressing different types of contamination [16]. The strategic combination of MoO_4_, Ag, and RGO takes advantage of the unique properties of each component, creating a highly efficient and sustainable solution for lead adsorption. This method not only boosts adsorption efficiency and versatility but also enhances the overall stability and reusability of the material, positioning it as a promising option for combating lead contamination in water resources. In this study, hydrothermally synthesized RGO-Ag2MoO_4_ NCs were utilized to eliminate Pb(II) from aqueous solutions. The adsorption process was optimized by adjusting various parameters, including the dosage of RGO-Ag_2_MoO_4_ NCs, pH levels, temperature, and the initial concentration of Pb(II). Additionally, kinetic, isotherm, and thermodynamic analyses were conducted to better understand the adsorption mechanism of Pb(II) onto the RGO-Ag_2_MoO_4_ NCs.

## 2. Results and Discussion

**Figure 1:**
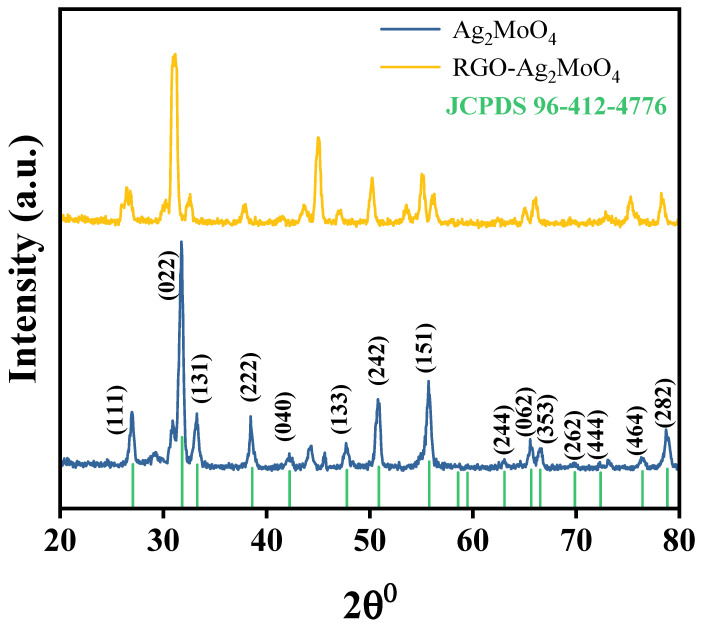
XRD of MoO_4_Ag_2_ and RGO-Ag_2_MoO_4_NCs.

The XRD pattern presented in Figure 1 shows the diffraction peaks of both pure Ag_2_MoO_4_ and the RGO-Ag_2_MoO_4_ composite, offering insights into the effect of RGO on the crystalline structure of Ag_2_MoO_4_. The blue curve, representing Ag_2_MoO_4_, exhibits sharp and well-defined peaks corresponding to crystal planes such as (111), (022), (131), and others, confirming a highly crystalline cubic phase, consistent with the JCPDS reference pattern (96-412-4776). In contrast, the yellow curve, representing the RGO-Ag_2_MoO_4_ NCs, shows a reduction in peak intensity and slight broadening. This indicates a decrease in crystallinity upon introducing RGO, which likely disrupts the regular packing of Ag_2_MoO_4_ crystallites, leading to smaller crystallite sizes or amorphous regions. Interestingly, there are no distinct peaks corresponding to RGO, suggesting that the RGO either has a low content in the composite or exists in an amorphous state, as typical RGO peaks around 24–26° might be too weak to be detected or overlap with Ag_2_MoO_4_ peaks. The reduced crystallinity, as evidenced by the broadened peaks, points to the successful integration of RGO into the Ag_2_MoO_4_ matrix, which may improve the composite’s conductivity and structural stability. RGO’s conductive properties could enhance the electrochemical performance of Ag_2_MoO_4_, making this composite potentially useful for applications in adsorption. The crystalline size (D) calculated using Debye-Scherrer equation $\left(D=\frac{0.9\lambda }{\beta cos\theta }\right)$ [17] was found to be 19.27 nm and 18.65 nm for Ag_2_MoO_4_ and RGO-Ag_2_MoO_4_ NCs, respectively. The results suggest that while the crystalline structure of Ag_2_MoO_4_ is retained in the composite, the interaction with RGO alters the crystallinity, potentially improving the composite’s functional properties.

**Figure 2:**
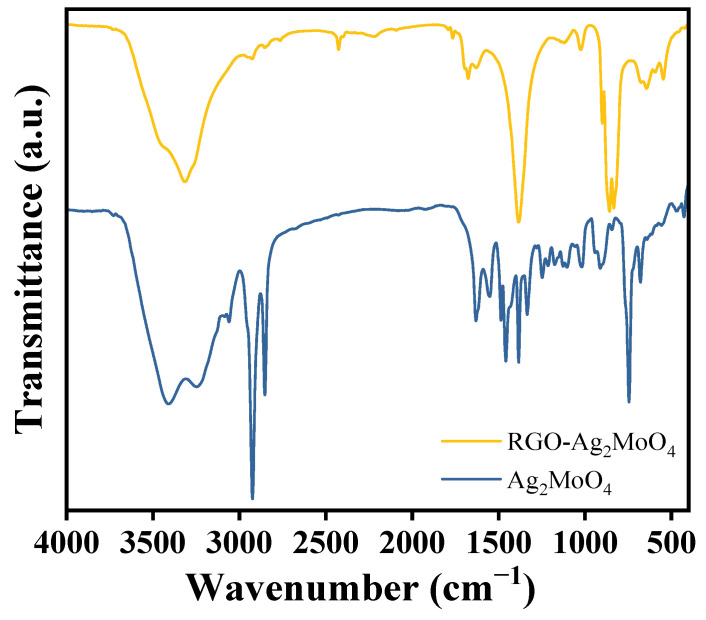
FTIR MoO_4_Ag_2_ and RGO-Ag_2_MoO_4_NCs.

The FTIR spectra (Figure 2) of Ag_2_MoO_4_ and RGO-Ag_2_MoO_4_ provide insights into the chemical interactions and structural changes upon incorporating RGO. In the Ag_2_MoO_4_ spectrum, characteristic peaks around 800–1000 cm^−1^ correspond to the Mo-O stretching vibrations, indicating the presence of molybdate (MoO_4_^2−^) groups [18]. Broad absorption bands near 3400 cm^−1^ is attributed to O-H stretching vibrations, signifying surface hydroxyl groups and moisture adsorption, while peaks around 1600–1700 cm^−1^ suggest the presence of adsorbed water molecules [19]. Upon integrating RGO, the FTIR spectrum of the RGO-Ag_2_MoO_4_ NCs shows reduced intensity in the O-H stretching region, indicating a decrease in moisture and surface hydroxyl groups. The appearance of a distinct peak around 1580 cm^−1^, corresponding to C=C stretching vibrations, confirms the presence of RGO’s graphitic structure [20]. Additionally, peaks near 1300–1400 cm^−1^ likely represent residual oxygenated groups in RGO, while the Mo-O peaks remain prominent, confirming that the molybdate structure is preserved. The preserved molybdate structure, as indicated by the Mo-O stretching peaks, ensures that the fundamental functionality of Ag_2_MoO_4_ is maintained. The FTIR data highlights the successful interaction between Ag_2_MoO_4_ and RGO, with the latter improving the material’s surface chemistry and functional properties.

**Figure 3:**
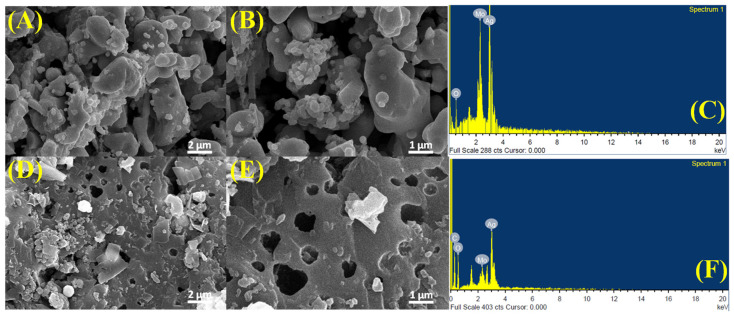
(**A**,**B**) SEM of MoO_4_Ag_2_, (**C**) Elemental mapping of MoO_4_Ag_2_, (**D**,**E**) SEM of RGO-Ag_2_MoO_4_NCs, (**F**) Elemental mapping of RGO-Ag_2_MoO_4_NCs.

The SEM and EDX analyses provide valuable insights into the morphological and compositional changes resulting from the incorporation of RGO into Ag_2_MoO_4_ (Figure 3A–F). In the SEM images of pure Ag_2_MoO_4_ (Figure 3A,B), the material displays a dense, clustered morphology with irregularly shaped particles forming large, aggregated structures. This dense configuration suggests a limited surface area, which could impede its performance in adsorption applications, where surface interactions are critical for effectiveness. The EDX spectra confirm the elemental composition of both samples, showing distinct peaks for silver (Ag), molybdenum (Mo), and oxygen (O), indicating the presence of Ag_2_MoO_4_ (Figure 3C). However, after the incorporation of RGO, significant morphological changes are observed. The SEM images of the RGO-Ag_2_MoO_4_ composite (Figure 3D,E) reveal a more open and porous structure, with visible voids and gaps between the Ag_2_MoO_4_ particles. This increased porosity, attributed to RGO acting as a scaffold, prevents particle agglomeration and promotes a more dispersed and structured arrangement. The enhanced porosity effectively increases the surface area, which is particularly advantageous for Pb(II) adsorption, as it provides more accessible sites for interaction with metal ions. In the RGO-Ag_2_MoO_4_ composite, the EDX spectra not only confirm the presence of Ag, Mo, and O (Figure 3F) but also show an additional peak for carbon (C), confirming the successful incorporation of RGO. This carbon peak corresponds to the reduced graphene oxide framework. One of the primary challenges in this study was maintaining uniform particle size and preventing agglomeration. Variability in nanoparticle size can significantly impact adsorption efficiency, as smaller particles typically offer a larger surface area for adsorption, thereby enhancing performance. However, synthesizing nanoparticles with a consistently narrow size distribution is inherently challenging due to factors such as material properties, which can result in agglomeration and, consequently, reduced accessibility of active sites. Addressing these challenges required careful control of parameters to optimize the stability and distribution of the nanocomposite structure, as even minor variations in size and morphology could lead to inconsistent adsorption performance across different batches.

**Figure 4:**
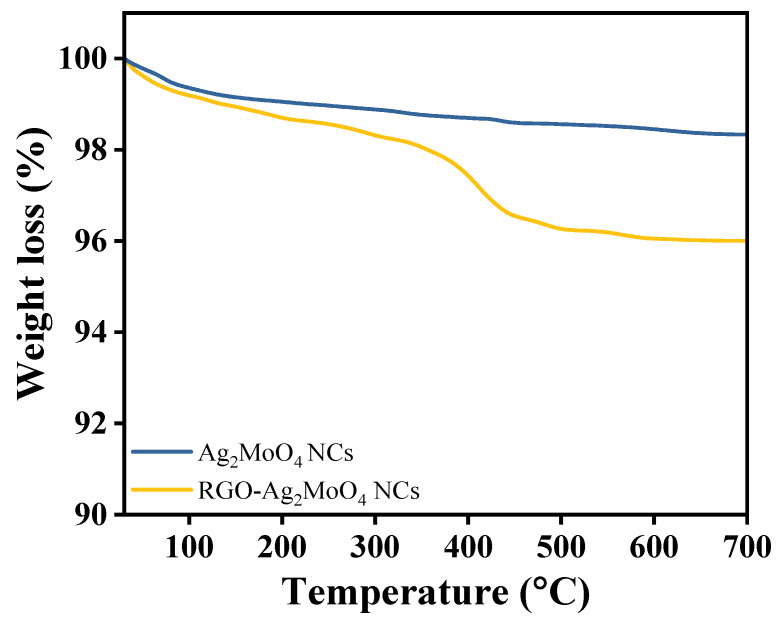
TGA curves of Ag_2_MoO_4_ NCs and RGO-Ag_2_MoO_4_ NCs.

The thermogravimetric analysis (TGA) plot provided reveals the thermal behavior of Ag_2_MoO_4_ and RGO-Ag_2_MoO_4_ across a temperature range from ambient to 700 °C (Figure 4). Initially, both samples exhibit minor weight loss below 200 °C, likely due to the evaporation of adsorbed moisture or other volatile species. This behavior is expected in materials with exposed surfaces, as adsorbed water or loosely bound molecules are released at relatively low temperatures. The weight loss for both materials in this region is minimal, indicating no significant organic impurities or other volatile contaminants. As the temperature increases from 200 °C to 400 °C, the Ag_2_MoO_4_ sample, represented by the blue line, shows a stable thermal profile with minimal additional weight reduction. This indicates that Ag_2_MoO_4_ alone has a high degree of thermal stability within this intermediate temperature range, remaining largely unaffected by thermal decomposition processes. In contrast, the RGO-Ag_2_MoO_4_ composite (yellow line) begins to display a gradual weight reduction over this same temperature range. This difference suggests that the presence of RGO introduces additional components or phases that are more susceptible to decomposition or oxidation as the temperature increases. The gradual weight loss in RGO-Ag_2_MoO_4_ could be attributed to the partial oxidation or degradation of carbon-based structures within the RGO layer, which typically occurs over a broad temperature range depending on the degree of reduction and structural integrity of the RGO. A more substantial weight loss is observed in the RGO-Ag_2_MoO_4_ sample around 400–500 °C. This prominent weight reduction is likely due to the decomposition or oxidation of the RGO component, which is more thermally labile compared to the metal oxide framework of Ag_2_MoO_4_. At elevated temperatures, RGO can undergo oxidation or structural breakdown, resulting in a notable loss in weight as carbon-based materials are more susceptible to thermal degradation. The pure Ag_2_MoO_4_ sample, on the other hand, remains stable through this temperature range, reflecting its robust thermal stability and indicating the absence of components susceptible to oxidation or decomposition at these temperatures. Above 500 °C, the weight loss in the RGO-Ag_2_MoO_4_ composite stabilizes, suggesting that most thermally degradable components in the composite have already decomposed. This stabilization indicates that any remaining structure, potentially the Ag_2_MoO_4_ core, retains its integrity up to 700 °C. The high-temperature stability of Ag_2_MoO_4_ throughout the entire temperature range highlights its intrinsic thermal resilience, making it more suitable for applications requiring stability at elevated temperatures. Incorporating RGO while potentially enhancing properties like surface area, electrical conductivity, or catalytic activity introduces a trade-off by reducing the composite’s thermal stability due to the thermal susceptibility of carbon-based RGO. This TGA analysis provides valuable insights into the thermal degradation patterns of both materials, underscoring Ag_2_MoO_4_s superior stability and the impact of RGO addition on the composite’s thermal endurance.

### Adsorption Study

Figure S1 displays the Pb(II) removal efficiency for the tested materials: RGO (63.88%), MoO_4_ (61.05%), Ag_2_MoO_4_ (70.47%), RGO-MoO_4_ (81.58%), and RGO-Ag_2_MoO_4_ (91.64%). A clear progressive increase in efficiency is observed, with RGO-Ag_2_MoO_4_ demonstrating the highest removal efficiency. The adsorption performance of MoO_4_ is attributed to its ability to provide active oxygen sites for Pb(II) binding. The introduction of Ag into the MoO_4_ structure enhances adsorption by creating additional active sites and increasing surface reactivity, which leads to improved Pb(II) capture. Furthermore, the addition of RGO into the composite significantly boosts the adsorption process by increasing the surface area available for Pb(II) ions and facilitating faster electron transfer. This synergy between RGO, Ag, and MoO_4_ contributes to the highest observed removal efficiency, underscoring the combined effect of increased active sites and enhanced electron mobility.

**Figure 5:**
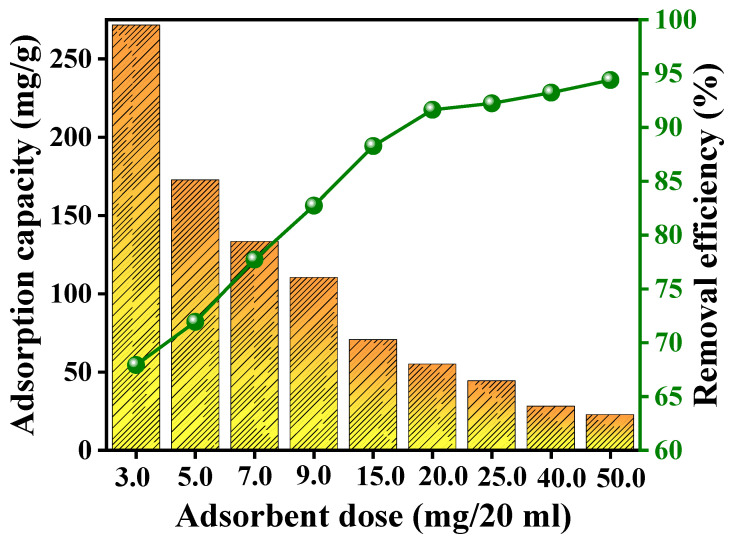
Effects RGO-Ag_2_MoO_4_ NCs dose on the adsorption of Pb(II).

To assess the influence of RGO-Ag_2_MoO_4_ NCs dose on the adsorption capacity and adsorption efficiency of Pb(II) ions, varying amounts of adsorbent (ranging from 3 mg/20 mL to 50 mg/20 mL) were tested (Figure 5). The outcomes presented an interesting trend: that as the adsorbent dose increased from 3 mg to 50 mg/20 mL, the removal efficiency of Pb(II) improved from 67.92 to 94.40%, while the adsorption capacity per unit mass of adsorbent showed a decreasing trend from 271.69 to 22.66 mg/g. This inverse relationship can be explained by the following factors: that as the adsorbent dose increases from 3 mg/20 mL to 20 mg/20 mL, the percentage of Pb(II) removed from the solution increases significantly—from 67.92 to 91.64%—due to the larger surface area and the availability of more adsorption sites. However, beyond the optimal dose of 20 mg/20 mL, further increases in the adsorbent dose did not yield a proportionate increase in removal efficiency, as the system approached equilibrium. At higher adsorbent doses, while more Pb(II) ions are removed from the solution overall, the adsorption capacity (amount of Pb(II) adsorbed per gram of adsorbent) decreases. This is because, at higher doses, more adsorption sites are available, but many of these sites remain unoccupied due to the limited amount of Pb(II) ions in the solution. Essentially, the ratio of available Pb(II) ions to the number of adsorption sites decreases, leading to a lower adsorption capacity. Thus, while removal efficiency increases with adsorbent dose, the adsorption capacity decreases owing to the saturation of available binding sites and the reduced concentration of Pb(II) ions per gram of sorbent [21]. The optimum adsorbent dose for the adsorption of Pb(II) onto the RGO-Ag_2_MoO_4_ NCs was chosen to be 20 mg/20 mL for further adsorption study.

**Figure 6:**
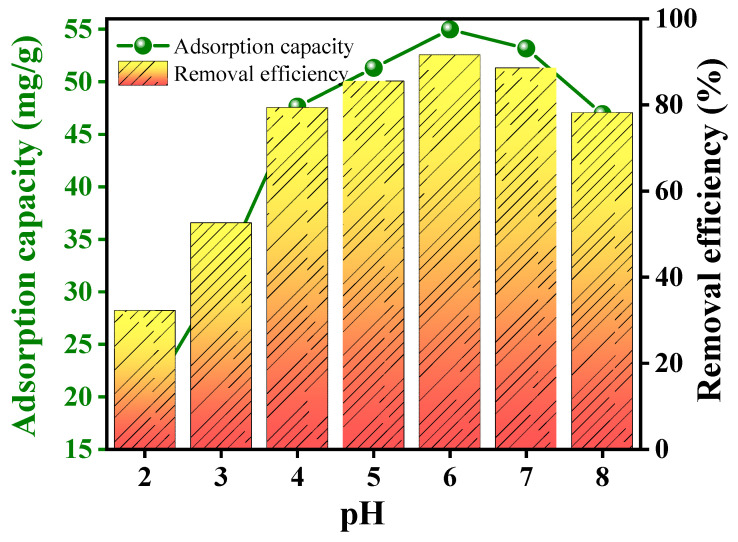
Effects on pH on the adsorption of Pb(II) using RGO-Ag_2_MoO_4_ NCs.

**Figure 7:**
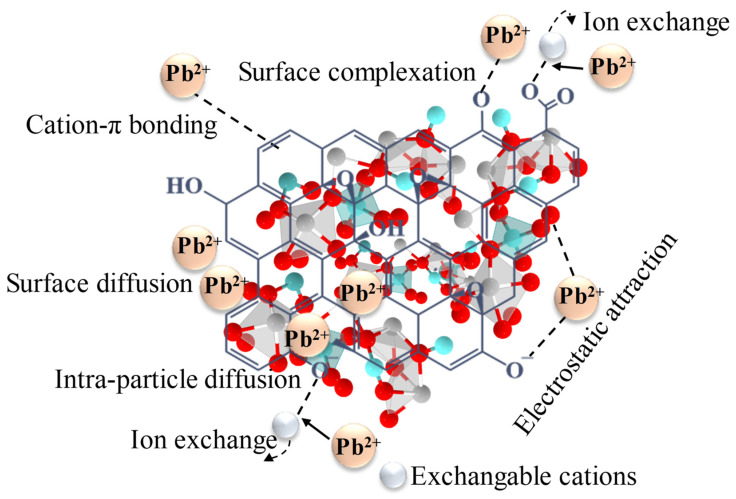
Possible interaction mechanisms for the adsorption of Pb(II) onto the RGO-Ag_2_MoO_4_ NCs.

**Figure 8:**
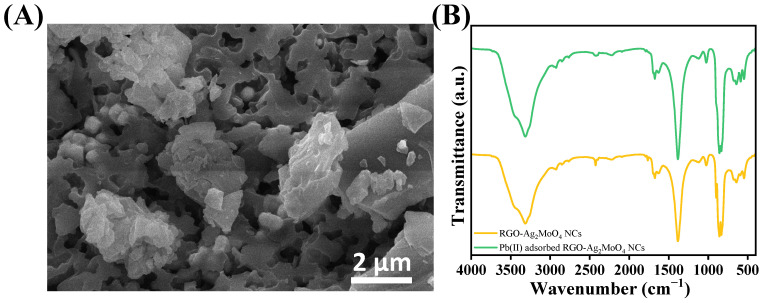
(**A**) SEM of RGO-Ag_2_MoO_4_ NCs after adsorption of Pb(II), (**B**) FTIR of RGO-Ag_2_MoO_4_ NCs before and after adsorption of Pb(II).

The pH of the solution plays a critical role in the adsorption process of heavy metals, influencing not only the surface charge of the adsorbent but also the speciation, mobility, and availability of the metal ions. The pH affects several key chemical processes, such as hydrolysis, complexation, redox reactions, and precipitation, all of which determine the efficiency of metal adsorption. In the case of lead (Pb(II)) adsorption onto RGO-Ag_2_MoO_4_ NCs, the optimal pH was found to be 6 with a removal efficiency of 91.64% (Figure 6). At lower pH levels, excessive protonation of the adsorbent surface occurs, which decreases the availability of binding sites for metal ions due to competition between protons (H^+^) and Pb(II) ions. As the pH increases, deprotonation of functional groups on the RGO-Ag_2_MoO_4_ NCs occurs, leading to a more negatively charged surface, which enhances the adsorption of positively charged Pb(II) ions. This trend continues until the pH reaches 6, at which maximum adsorption was achieved. Beyond pH 6, the adsorption efficiency declines due to the onset of lead hydroxide precipitation (e.g., Pb(OH)_2_), which reduces the availability of free Pb(II) ions for adsorption. Therefore, the ideal pH for Pb(II) removal using RGO-Ag_2_MoO_4_ NCs was 6, where optimal sorption was achieved due to favorable electrostatic interactions and minimized competition with protons. This pattern aligns with studies showing that the adsorption of lead is highly dependent on pH, as observed in other sorbents [22,23]. The surface of RGO-Ag_2_MoO_4_ NCs likely contains active sites or functional groups that can attract and bind Pb(II) ions through different chemical interactions, such as electrostatic attraction, complexation, or ion exchange as shown in Figure 7. The SEM images taken after Pb(II) adsorption (Figure 8A) reveal that the RGO-Ag_2_MoO_4_ NCs surface has become less porous compared to the SEM image of RGO-Ag_2_MoO_4_ NCs before adsorption (Figure 3D,E), suggesting that Pb(II) ions effectively occupy the available active sites and surface voids. Before adsorption, the nanocomposite displayed an open, porous structure that provided ample surface area and accessible sites for interaction. However, following Pb(II) adsorption, the reduction in visible porosity indicates that the Pb(II) ions are now covering these surface pores. The FTIR analysis of the RGO-Ag_2_MoO_4_ NCs before and after Pb(II) adsorption (Figure 8B) reveals significant changes in functional groups, suggesting specific interactions between Pb(II) ions and the composite surface. Initially, a broad O-H stretching band is observed around 3400 cm^−1^, indicating surface hydroxyl groups. After Pb(II) adsorption, the intensity of this band increases, implying that hydroxyl groups are actively involved in binding Pb(II) ions, likely through hydrogen bonding or ion-dipole interactions. This change suggests that these groups serve as primary active sites for Pb(II) capture. Additionally, the C=C stretching vibrations at approximately 1580 cm^−1^, characteristic of the RGO structure, exhibit a shift or slight change in intensity post-adsorption, indicating potential cation-π interactions between Pb(II) ions. Furthermore, oxygen groups on RGO, indicated by peaks around 1300–1400 cm^−1^, also show changes, suggesting that these groups may participate in complexation or ion exchange with Pb(II) ions. Meanwhile, the Mo-O bonds within the molybdate structure (typically found around 800–1000 cm^−1^) remain largely stable after adsorption, implying that the core molybdate structure is preserved. However, slight shifts in these peaks hint at minor interactions between Pb(II) ions and the molybdate surface. Collectively, these FTIR changes confirm that the RGO-Ag_2_MoO_4_ NCs binds Pb(II) ions through various interaction mechanisms, underscoring the composite’s effectiveness for Pb(II) adsorption in aqueous solutions.

**Figure 9:**
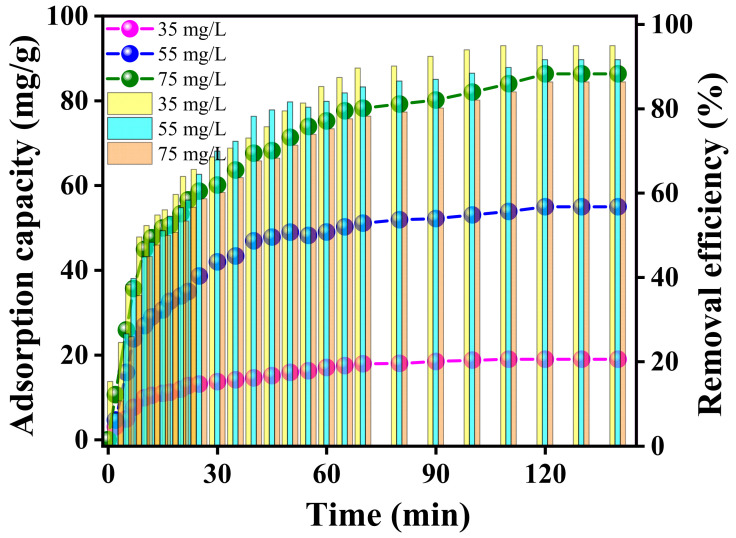
Effect of time on the adsorption of Pb(II) adsorption using RGO-Ag_2_MoO_4_ NCs.

The adsorption of Pb(II) ions onto RGO-Ag_2_MoO_4_ NCs was investigated by analyzing the effects of contact time (ranging from 2 to 140 min) and varying initial lead concentrations. The results, as shown in Figure 9, indicate that the amount of Pb(II) adsorbed increased progressively with time, reaching equilibrium at around 120 min for all tested concentrations. The adsorption process followed a two-phase pattern: an initial rapid stage, during which around 45% of the lead ions were adsorbed at all concentrations within the first 10 min, followed by a slower phase as equilibrium approached. This adsorption behavior can be attributed to the availability of active sites on the RGO-Ag_2_MoO_4_ NCs surface. During the early phase, these sites were highly accessible, enabling a rapid adsorption process. However, as time progressed, the active sites became increasingly saturated, and competition among the remaining Pb(II) ions for the limited available sites slowed the adsorption rate. This slowdown explains the transition to the slower phase until equilibrium was reached after 120 min, a pattern also observed by Mahmood et al. [24]. They noted that enhanced accessibility to active sites renders the early stage swift, but over time, saturation and ion competition delay the process. Despite the increase in initial lead concentration, the equilibrium time remained consistent at 120 min across all concentrations. This can be explained by the fact that with higher lead concentrations, more Pb(II) ions were present in the solution, creating a stronger driving force for the diffusion of lead ions toward the adsorbent surface. This greater concentration gradient accelerated the adsorption process, owing to a rise in the adsorption capacity as more ions were able to interact with the available active sites. However, the adsorbent’s active sites reached saturation within the same time frame, as the equilibrium adsorption capacity is dependent on the number of available sites rather than solely on concentration. Thus, even with higher initial lead concentrations, equilibrium was reached at the same time—120 min, due to the finite number of adsorption sites on the RGO-Ag_2_MoO_4_ NCs. Based on these observations, 120 min was considered the optimum contact time for efficient Pb(II) removal across various lead concentrations.

**Figure 10:**
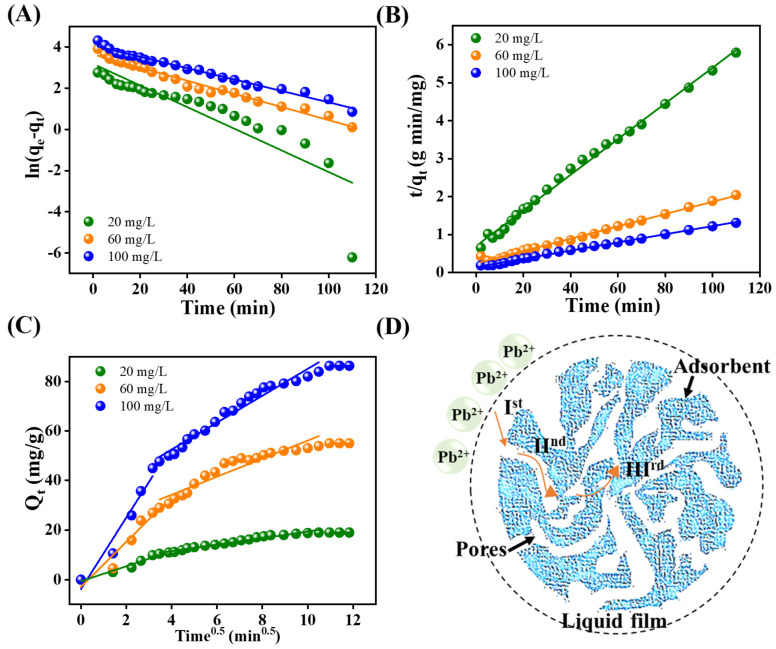
Linear kinetics graphs: (**A**) Psuedo-first-order; (**B**) Psuedo-second-order; (**C**,**D**) IPD model and pictorial representation of Pb(II) diffusion into the RGO-Ag_2_MoO_4_ NCs (I^st^ (surface diffusion), II^nd^ (pore diffusion), and III^rd^ (equilibrium approaching stage).

The adsorption kinetics of b(II) ions onto RGO-Ag_2_MoO_4_ NCs were analyzed using various kinetic models, including the pseudo-first-order, pseudo-second-order, and Weber–Morris intraparticle diffusion models (IPD), to understand the adsorption mechanism and controlling factors as shown in Figure 10A–C. The equations of these models are given in the Supplementary File Equations (S3)–(S5) in Text S3. Among these models, the pseudo-second-order model provided the best fit for the experimental data, suggesting that chemisorption plays a key role in the adsorption process. The pseudo-first-order kinetic model, commonly used to describe physical adsorption processes, was applied to the data. However, the correlation coefficients (R^2^) obtained from this model were low, and the calculated equilibrium adsorption capacities (q_e_) significantly deviated from the experimentally observed q_e_ values (Table 1). This indicates that the pseudo-first-order model is inadequate for describing the interactions between Pb(II) ions and the RGO-Ag_2_MoO_4_ NCs. In contrast, the pseudo-second-order kinetic model, which assumes that the rate-limiting step involves chemisorption—where chemical bonding through electron sharing or exchange occurs between the adsorbent and the adsorbate—proved to be much more accurate. The plot of t/q_t_ versus t resulted in straight lines for all lead concentrations tested, with high correlation coefficient values (R^2^ > 0.99) (Figure 10B and Table 1). This strong linearity and the close agreement between the calculated and experimental q_e_ values confirmed that the adsorption of Pb(II) onto the RGO-Ag_2_MoO_4_ NCs follows pseudo-second-order kinetics. This indicates that the adsorption process is primarily controlled by chemical interactions, such as the formation of chemisorptive bonds between Pb(II) ions and the functional groups on the NCs surface.

The Weber–Morris intraparticle diffusion model (IPD), depicted in Figure 10C,D, outlines three distinct stages in the adsorption process: (I) Pb(II) ions first diffuse onto the adsorbent’s surface; (II) the ions then penetrate deeper into the material’s porous structure; (III) equilibrium is eventually reached, resulting in the saturation of the available adsorption sites [25]. The model’s findings show that the initial phase (k_i1_) exhibits the fastest adsorption rate, which progressively declines during the next stage (k_i2_) before reaching equilibrium (k_i3_). This trend emphasizes that the adsorption process begins rapidly and slows down as it progresses toward equilibrium.

**Figure 11:**
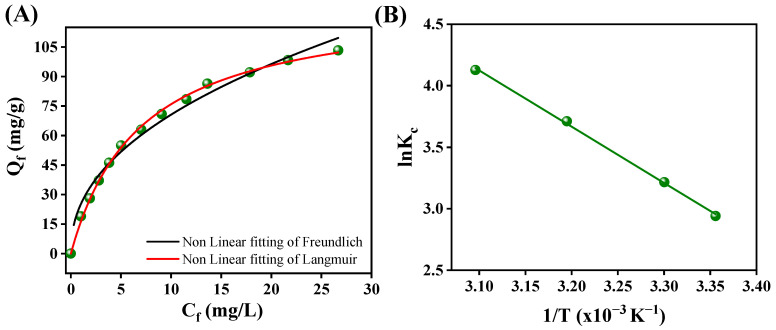
(**A**) Non-linear graph of Langmuir and Freundlich models; (**B**) lnK_c_ vs. 1/T plot.

The adsorption data for Pb(II) ions onto RGO-Ag_2_MoO_4_ NCs were analyzed using the Langmuir and Freundlich isotherm models to better understand the adsorption mechanism and capacity. The equations of these models are given in the Supplementary File Equations (S6) and (S7) Text S3. The results demonstrated that the Langmuir isotherm model provided the best fit for the experimental data, as indicated by a higher correlation coefficient (R^2^ = 0.998) and a strong degree of linearity (Figure 11A and Table 2). This confirms that the Pb(II) adsorption onto RGO-Ag_2_MoO_4_ NCs is consistent with monolayer adsorption on the surface of the nanocomposite, with a theoretical maximum adsorption capacity (Q_L_) for Pb(II) ions of 128.94 mg/g. This suggests that each Pb(II) ion occupies a specific adsorption site without significant interaction between adsorbed ions. In contrast, the Freundlich isotherm model exhibited a lower correlation coefficient (R^2^ = 0.976), indicating that it was less suitable for describing the adsorption of Pb(II) onto RGO-Ag_2_MoO_4_ NCs. The Freundlich model accounts for multilayer adsorption and surface heterogeneity, which did not accurately capture the adsorption behavior observed in this study. The adsorption capacity was found to be higher in comparison with the existing literature given in Table 3 [26,27,28,29,30,31,32]. To assess the favorability of the adsorption process, the dimensionless separation factor or equilibrium parameter (R_L_) was calculated by $R_{L}=\frac{1}{1+K_{L}C_{o}}$ [33]. The R_L_ values were found to be within the range of 0 to 1, which indicates that the adsorption of Pb(II) ions onto RGO-Ag_2_MoO_4_ NCs was favorable.

The influence of system temperature on the removal of Pb(II) using RGO-Ag_2_MoO_4_ NCs was examined, with the corresponding fitting curves and data shown in Figure S2. As the temperature increased from 298 K to 323 K, the removal efficiency for Pb(III) improved from 91.64% to 97.63%, suggesting that higher temperatures enhanced the adsorption process. The heat of sorption (ΔH), Gibbs energy change (ΔG), and entropy change (ΔS) of the sorption of Pb(II) onto RGO-Ag_2_MoO_4_ NCs were calculated using Equations (S8) and (S9) in Text S3. Thus, from ln K_c_ versus 1/T plot (Figure 11B), the ΔH is calculated and found to be 37.99 kJ/mol. A positive value of ΔH confirms the endothermic nature of the process. The negative values of ΔG (Table 4) indicate the spontaneous nature of sorption for Pb(II). The positive values of ΔS (152.07 J/K/mol) suggest the increased randomness at the solid/solution interface during the removal of Pb(II) onto RGO-Ag_2_MoO_4_ NCs [34].

**Figure 12:**
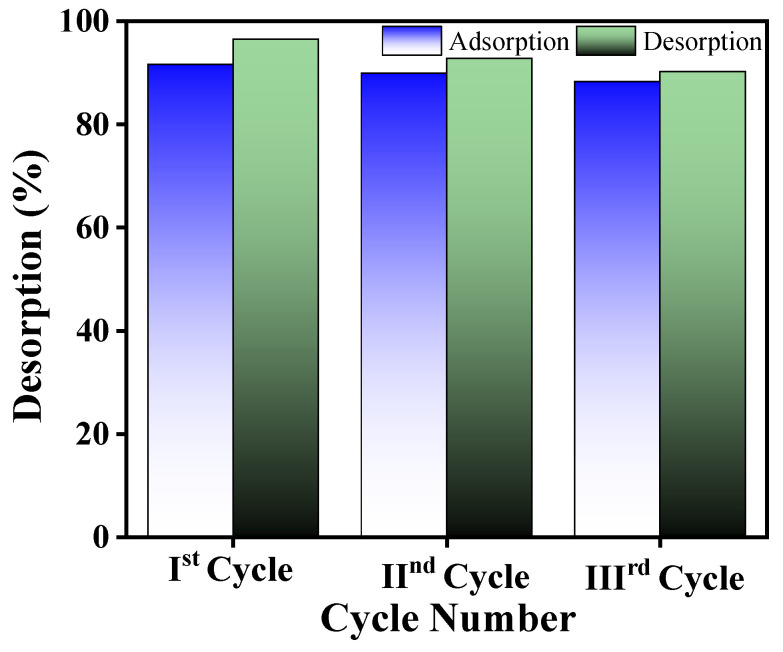
Adsorption and desorption performance in the cycling experiment.

Desorption experiments were performed by agitating Pb(II)-loaded RGO-Ag_2_MoO_4_ NCs with 0.1 M HCl, 0.1 M NaOH, C_2_H_5_OH and distilled water and equilibrates for 120 min and then centrifuged. After centrifugation, the supernatant was analyzed and percent desorption (%D) was computed by employing the following relation [35]:

(1) $$\%D=\frac{m_{d}}{m_{a}}\times 100$$

where, m_a_ and m_d_ represent the concentrations of Pb(II) ion adsorbed and desorbed in mg/L, respectively. The results showed that the desorption efficiency remained above 90% for up to three adsorption–desorption cycles. Importantly, the adsorption capacity of the nanocomposite decreased only by 3.28% after the third cycle, indicating strong reusability and minimal loss in performance over multiple cycles (Figure 12).

**Figure 13:**
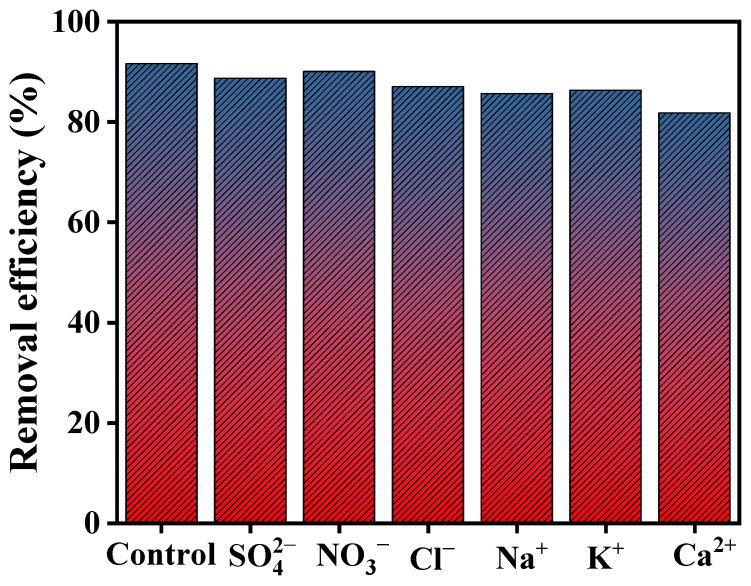
Effect of co-existing ions on Pb(II).

To evaluate the practical applicability of the synthesized RGO-Ag_2_MoO_4_ NCs in real-world wastewater treatment, a selectivity test was conducted to investigate the effect of co-existing ions commonly found in wastewater. These ions included both anions (Cl^−^, NO_3_^−^, and SO_4_^2−^) and cations (Na^+^, K^+^, and Ca^2+^). The performance of the nanocomposite in the presence of these ions was evaluated under optimal conditions (Figure 13). The results showed that the anions had a negligible effect on the adsorption performance of the RGO-Ag_2_MoO_4_ NCs, with no significant reduction in Pb(II) removal efficiency observed. However, the presence of cationic species, especially Ca^2+^, had a more pronounced negative impact on the adsorption efficiency [36]. This is likely due to the competitive binding between the cationic species and Pb(II) ions for the available active sites on the nanocomposite surface. The greater negative effect of Ca^2+^ is attributed to its lower ionic radius, which allows for easier competition with Pb(II) ions. This finding is consistent with previous reports [36]. Despite this competitive interaction, the RGO-Ag_2_MoO_4_ NCs retained a high lead adsorption capacity, highlighting its potential for the selective removal of Pb(II) even in the presence of common co-existing ions found in wastewater.

## 3. Experimental Section

### Synthesis of Reduced Graphene Oxide Modified Silver Molybdate Ternary Nanocomposite (RGO–Ag_2_MoO_4_ NCs)

The synthesis of RGO-Ag_2_MoO_4_ nanocomposites (NCs) involves the following detailed steps:

Step 1: Preparation of Graphene Oxide (GO)

Graphite is oxidized to graphene oxide (GO) using the modified Hummer’s method [37]. This method combines concentrated sulfuric acid (100 mL), sodium nitrate (1 g), and potassium permanganate (12 g) with graphite (2 g). This reaction exfoliates graphite into hydrophilic GO sheets by introducing oxygen functional groups (hydroxyl, epoxide, and carboxyl groups) onto the carbon basal planes, enhancing GO’s dispersibility in water.

The simplified reactions are as follows:

Oxidation Reaction:

$$C\;+\;{KMnO}_{3}\;+\;H_{2}{SO}_{4}\;\;\to \;\;GO\;+\;{MnO}_{4}^{-}$$

Functionalization:

Various functional groups are attached to the GO sheets through further reactions with sulfuric acid (H_2_SO_4_) and other reagents, introducing groups such as –OH, −COOH and −C–O–C.

Step 2: Preparation of Precursor Solution

Sodium molybdate (1 mM) and silver nitrate (1 mM) are separately prepared and introduced into an aqueous GO suspension. Here, sodium molybdate dissociates to provide MoO_4_^2−^ ions, and silver nitrate dissociates to provide Ag^+^ ions. In the subsequent steps, these ions serve as essential precursors for forming Ag_2_MoO_4_ nanoparticles. The sodium molybdate and silver nitrate concentration is typically set to 1 mM, while the GO concentration is adjusted to 2 mg/mL in the solution.

Step 3: Hydrothermal Treatment

The precursor solution undergoes hydrothermal treatment at 180 °C for 24 h under alkaline conditions (pH~9). During this treatment:

GO is reduced to reduced graphene oxide (RGO) as oxygen-containing groups are partially removed, restoring the sp^2^ carbon structure and enhancing its conductivity.

Ag^+^ ions react with MoO_4_^2−^ ions, leading to the nucleation and growth of Ag_2_MoO_4_ nanoparticles, which anchor onto the RGO surface. The residual oxygen functional groups on RGO, such as hydroxyl and carboxyl groups, facilitate the attachment of Ag_2_MoO_4_ nanoparticles via electrostatic attractions and hydrogen bonding.

In the aqueous suspension, sodium molybdate (Na_2_MoO_4_) and silver nitrate (AgNO_3_) dissociate, providing ${MoO}_{4}^{2-}$ and ${Ag}^{+}$ ions, respectively.

Dissociation:

$${Na}_{2}{MoO}_{4}\;\;\to \;\;2{Na}^{+}+{MoO}_{4}^{2-}$$

$${AgNO}_{3}\;\;\to \;\;{Ag}^{+}\;+\;{NO}_{3}^{-}$$

Formation of Ag_2_MoO_4_:

Under alkaline hydrothermal conditions, ${Ag}^{+}$ ions react with ${MoO}_{4}^{2-}$ ions to form Ag_2_MoO_4_ nanoparticles

$${2Ag}^{+}+\;{MoO}_{4}^{2-}\;\;\to \;\;{Ag}_{2}{MoO}_{4}$$

Reduction of GO to RGO and Formation of RGO-Ag_2_MoO_4_ Nanocomposite

During the hydrothermal process at 180 °C, GO is partially reduced to reduced graphene oxide (RGO), as some oxygen-containing functional groups are removed, restoring the sp^2^ carbon structure. Simultaneously, Ag_2_MoO_4_ nanoparticles anchor onto the RGO sheets due to residual oxygen functionalities (hydroxyl and carboxyl groups) through electrostatic interactions and hydrogen bonding.

$$GO+\;H^{+}+\;e^{-}\;\;\;\to \;\;RGO+\;H_{2}O$$

The Ag_2_MoO_4_ nanoparticles interact with the RGO surface through electrostatic interactions and possible covalent bonding with remaining oxygen-containing functional groups, forming a stable RGO-Ag_2_MoO_4_ nanocomposite.

$$GO+{Na}_{2}{MoO}_{4}+{AgNO}_{3}+H_{2}O\;\to \;\;\;\;RGO-{Ag}_{2}{MoO}_{4}+\;{NaNO}_{3}$$

Step 4: Formation of RGO-Ag_2_MoO_4_ Nanocomposite

**Figure 14:**
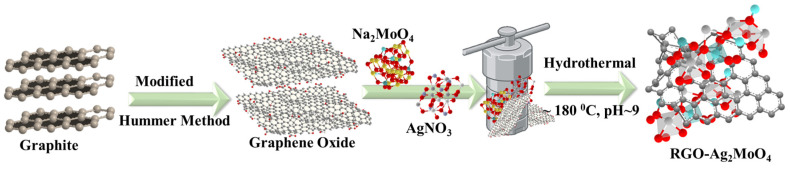
Synthesis scheme of RGO-Ag_2_MoO_4_NCs.

The hydrothermal process yields a stable nanocomposite with Ag_2_MoO_4_ nanoparticles uniformly dispersed on the RGO surface. This distribution prevents the aggregation of nanoparticles and enhances the composite’s structural stability and surface area, which are beneficial for applications in adsorption. The resulting RGO-Ag_2_MoO_4_ NCs exhibit synergistic properties, combining the high surface area and conductivity of RGO with the active adsorption sites of Ag_2_MoO_4_, making it practical for environmental remediation, particularly for the removal of heavy metals like Pb(II) from wastewater. The Schematic synthesis procedure of RGO-Ag_2_MoO_4_ NCs is given in Figure 14.

## 4. Conclusions

This study highlights the effective utilization of RGO-Ag_2_MoO_4_ NCs, synthesized via a hydrothermal process, as efficient adsorbents for the removal of Pb(II) ions from aqueous solutions. Several key factors influence the adsorption efficiency of the RGO-Ag_2_MoO_4_ NCs, including adsorbent dosage, contact time, solution pH, initial Pb(II) concentration, and system temperature. The RGO-Ag_2_MoO_4_ NCs demonstrated remarkable adsorption performance, achieving a removal efficiency approximately in the range of 94.96% to 86.37% for Pb(II) concentrations between 20 and 100 mg/L at a solution pH of 6, and an equilibrium time of 120 min. According to the Langmuir isotherm model, Pb(II) ions formed a monolayer on the surface of the RGO-Ag_2_MoO_4_ NCs, with a maximum adsorption capacity of 128.94 mg/g. The time-dependent experimental data aligned well with the pseudo-second-order kinetic model, indicating a strong correlation between adsorption rate and time. The potential adsorption mechanisms for Pb(II) removal include surface precipitation, ion exchange, electrostatic attraction, and physical adsorption. To further assess the practical applicability of RGO-Ag_2_MoO_4_ NCs, a selectivity test was performed in real wastewater, investigating the effect of co-existing ions such as anions (Cl^−^, NO_3_^−^, and SO_4_^2−^) and cations (Na^+^, K^+^, and Ca^2+^). The results showed that anions had a negligible effect on the adsorption performance, while cations, especially Ca^2+^, had a more pronounced impact due to competitive binding. Despite this, the material maintained a high lead adsorption capacity, indicating its robustness for selective Pb(II) removal even in complex wastewater environments. Overall, this study concludes that RGO-Ag_2_MoO_4_ NCs are highly effective adsorbents with excellent adsorption characteristics and significant capacity. Their ability to effectively remove Pb(II) ions from contaminated water, combined with their stability under varying conditions, positions them as a promising candidate for large-scale wastewater treatment and environmental remediation applications.

## Figures and Tables

**Table 1:** Kinetic parameters for removal of Pb(II) onto RGO-Ag_2_MoO_4_ NCs (Experimental parameters: RGO-Ag_2_MoO_4_ NCs dose = 20 mg/20 mL, pH = 6, temperature = 298 K).

Kinetics	Parameters			
Experimental	Co (mg/L)	20	60	100
	Q_exp_ (mg/L)	18.992	54.983	86.372
Pseudo-first-order	Q_fo_ (mg/g)	24.312	38.474	59.781
	K_1_ (g/mg min)	0.0469	0.0162	−0.0277
	R^2^	0.772	0.979	0.983
Pseudo-second-order	Q_so_ (mg/g)	21.321	61.666	92.981
	K_2_ (g/mg min)	3.136 × 10^−3^	1.079 × 10^−3^	4.4056 × 10^−5^
	R^2^	0.996	0.990	0.996
Weber–Morris intraparticle diffusion model	K_i,1_ (mg/g min^−1/2^)	3.043	9.081	14.382
	R^2^	0.945	0.914	0.949
	K_i,2_ (mg/g min^−1/2^)	1.297	3.651	5.446
	R^2^	0.972	0.901	0.967
	K_i,3_ (mg/g min^−1/2^)	0.00228	0.00341	0.00341
	R^2^	0.998	0.960	0.960

**Table 2:** Isotherm model factors for the elimination of Pb(II) onto RGO-Ag_2_MoO_4_ NCs (Experimental parameters: RGO-Ag_2_MoO_4_ NCs dose = 20 mg/20 mL, pH = 6, temperature= 298 K, time = 120 min).

Isotherms	Parameters	Pb(II)
Langmuir	Q_L_ (mg/g)	128.942
	K_L_ (L/g)	0.1432
	R^2^	0.9982
Freundlich	K_F_ (mg/g)	25.151
	n_F_ (L/g)	0.448
	R^2^	0.9766

**Table 3:** Comparison with the reported literature data for the adsorption of Pb(III) onto the various adsorbents.

Adsorbents	Adsorption Capacity (mg/g)	Dose (g/L)	pH	Time (h)	Temperature (K)	Ref.
RGO-Fe^0^/Fe_3_O_4_-PEI	60.24	0.05	6	1	268	[26]
GO/Fe_3_O_4_	38.5	-	6	2	293	[27]
Carbon/iron oxide	67.1	2	6	1	323	[28]
GO-polydopamine	53.6	-	-	5	302	[29]
TiO_2_/rGO	9.1	1	-	2	-	[30]
Fe_3_O_4_@SiO_2_-MnO_2_	35.1	0.5	4	24	298	[31]
ZnO/Carbon nanofibers	92.59	1	7	45 min	-	[32]
RGO-Ag_2_MoO_4_ NCs	128.94	1	6	2	298	Our result

**Table 4:** Thermodynamic factors for the removal of Pb(II) onto RGO-Ag_2_MoO_4_ NCs (Experimental parameters: RGO-Ag_2_MoO_4_ NCs dose = 20 mg/20 mL, pH = 6, concentration of Pb(II) = 60 mg/L).

T(K)	∆S° (J/K/mol)	∆H° (kJ/mol)	∆G° (kJ/mol)	R^2^
298	152.068	37.995	−7.286	0.9989
303			−8.101	
313			−9.656	
323			−11.083	

## Data Availability

Data are contained within the article.

## Supplementary Materials

The following supporting information can be downloaded at: https://www.mdpi.com/article/10.3390/molecules29215152/s1, Text S1: Materials; Text S2: Instrumental Characterization; Text S3: Batch adsorption studies; Figure S1: Removal efficiency of Pb(II) onto the RGO, MoO_4_, Ag_2_MoO_4_, RGO-MoO_4_ and RGO-Ag_2_MoO_4_ NCs; Figure S2: Effect of temperature on the removal efficiency of Pb(II) onto the RGO-Ag_2_MoO_4_ NCs [38,39].
