# Supplementary material for: A Facile Synthesis of RGO-Ag2MoO4 Nanocomposites for Efficient Lead Removal from Aqueous Solution
Source: Molecules. 2024 Oct 31;29(21):5152. doi: 10.3390/molecules29215152 (PMC11547552; doi:10.3390/molecules29215152)
Supplement: Supplementary file 1 [file molecules-29-05152-s001.zip › molecules-3277649-supplementary.pdf]

A Facile Synthesis of RGO-Ag_2MoO_4 Nanocomposites for Efficient Lead Removal from Aqueous Solution

Mohd Shoeb †, Fouzia Mashkoor †, Mohmmad Naved Khan and Changyoon Jeong *

School of Mechanical Engineering, Yeungnam University, Gyeongsan 38541, Republic of Korea; mshoeb@yu.ac.kr (M.S.); fmashkoor@yu.ac.kr (F.M.); navedkhan@yu.ac.kr (M.N.K.)

* Correspondence: yoonni22@yu.ac.kr

† These authors contributed equally to this work.

1S. Materials

All the chemical reagents used in this research work were of analytical reagent (AR) grade from from Sigma Aldrich, South Korea.

2S. Instrumental Characterization

The adsorbent was examined by FTIR, SEM, and XRD techniques. The FTIR spectra from 4000 to 400 cm^{-1} were recorded for the analyses of functional groups using Nicolet IS50 Thermo Fisher Scientific FTIR Spectrometer. Surface morphology were examined by using Scanning Electron Microscope (SEM, JEOL, JSM6510LV, Japan). The crystallinity of the composite was studied by X-ray Powder Diffraction (MiniFlex™ II benchtop XRD system, Rigaku Corporation, Tokyo, Japan) in the 2θ range of 10–80° at 40 kV and a current of 30 mA.

3S. Batch adsorption studies

The RGO-Ag_2MoO_4 NCs as an adsorbents for Pb(II) ion removal experiments were performed as follow. Typically, 20 mg of the RGO-Ag_2MoO_4 NCs were mixed with 20.0 mL of 60 mg/L concentration of Pb(II) solution at room temperature. The initial pH value of Pb(II) solution was adjusted to 6. At pre-set intervals, a certain amount of the suspension was withdrawn from the system. The supernatant obtained from different intervals was monitored by using Inductively Coupled Plasma-Atomic Emission Spectroscopy, ICP-AES (OPTIMA 8300, Perkin Elmer) to confirm the residual Pb(II) in the system. The adsorbed amount (q_t/q_e) onto the RGO-Ag_2MoO_4 NCs was determined from the change in the concentrations of Pb(II) at any time/equilibrium (t/e) by using following equation (Equation S1):

$$q_{t/e} = \left(C_o - C_{t/e}\right) \times \frac{V}{m} \qquad \text{(S1)}$$

The removal efficiency (%R) was obtained by using the following expression (Equation S2):

$$\%\mathrm{R} = \frac{(\mathrm{C_0} - \mathrm{C_{t/e}})}{\mathrm{C_0}} \times 100 \tag{S2}$$

where, $C_o/C_t/C_e$ (mg/L) signifies the initial/any time/equilibrium liquid-phase concentrations of solute, respectively.

The linear forms of the P-fo, P-so, and I-D models are described in Equations S3-S5 [1,2]. In these equations, k_1, k_2, and k_3 are the equilibrium rate constants of the P-fo, P-so, and I-D models, respectively, while Q_{fo} and Q_{so} represent the equilibrium adsorption capacities (mg/g) of the P-fo and P-so models, respectively. C is a constant.

P-fo: $$\ln(Q_f - Q_t) = lnQ_{f_o} - K_1 t \tag{S3}$$

P-so: $$\frac{t}{Q_t} = \frac{1}{K_2 Q_{so}^2} + \frac{t}{Q_{so}} \tag{S4}$$

I-D: $$Q_t = K_3 t^{0.5} + C \tag{S5}$$

The experimental data was fitted using two adsorption isotherms models namely, Langmuir, and Freundlich models to theoretically explore the adsorption process. The isotherm models are expressed in Equations S6-S7. All adsorption isotherm models were used by the nonlinear form of Q_f versus C_f. Here, Q_L, is a maximum adsorption capacity (mg/g) of Langmuir. Meanwhile, K_L (L/mol), and K_F (L/mg) respectively, are the constants of Langmuir, and Freundlich models, which are related to the adsorption capacity or the interaction between the adsorbent and the adsorbate. Besides, n_F is a constant of Freundlich expressions, which represent the adsorption intensity. R (8.314 J mol/K), and T, are the universal gas constant, and temperature in Kelvin, respectively.

$$Q_f = \frac{Q_L K_L C_f}{1+K_L C_f} \quad \text{(S6)}$$

$$Q_f = K_F C_f^{\frac{1}{n_F}} \quad \text{(S7)}$$

In these isotherms, the Langmuir model (Equation S6) and the empirical Freundlich formula (Equation S7) are the monolayer sorption on the uniform surface and multilayer sorption on the heterogeneous surface, respectively.

The thermodynamic parameters i.e., Gibb's free energy (ΔG), enthalpy (ΔH), and entropy (ΔS) were computed by the Equation S8 and S9

$$\Delta \mathrm{G} = -\mathrm{RTlnK}_c \quad \text{(S8)}$$

$$\mathrm{lnK}_c = \frac{-\Delta \mathrm{G}}{\mathrm{RT}} = \frac{\Delta \mathrm{S}}{\mathrm{R}} - \frac{\Delta \mathrm{H}}{\mathrm{RT}} \quad \text{(S9)}$$

where, K_c is the thermodynamic constant and can be estimated by the ratio of adsorption capacity to the residual concentration of metal ion molecule in the solution.

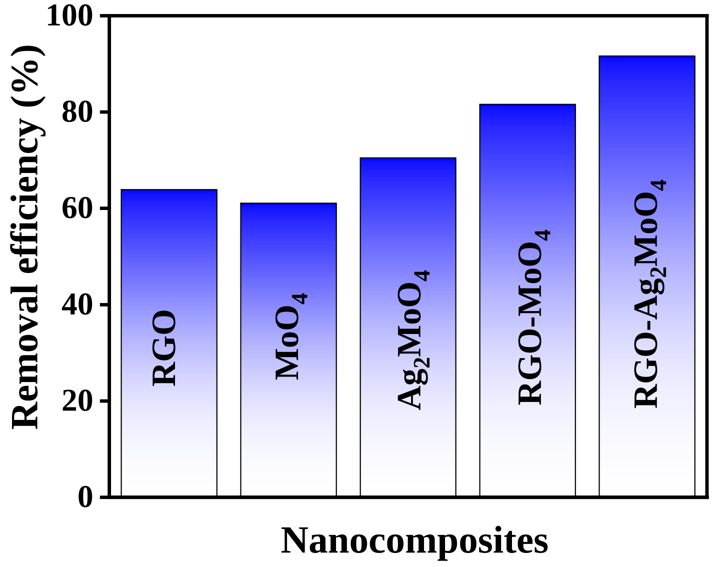

Figure S1. Removal efficiency of Pb(II) onto the RGO, MoO_4, Ag_2MoO_4, RGO-MoO_4 and RGO-Ag_2MoO_4 NCs.

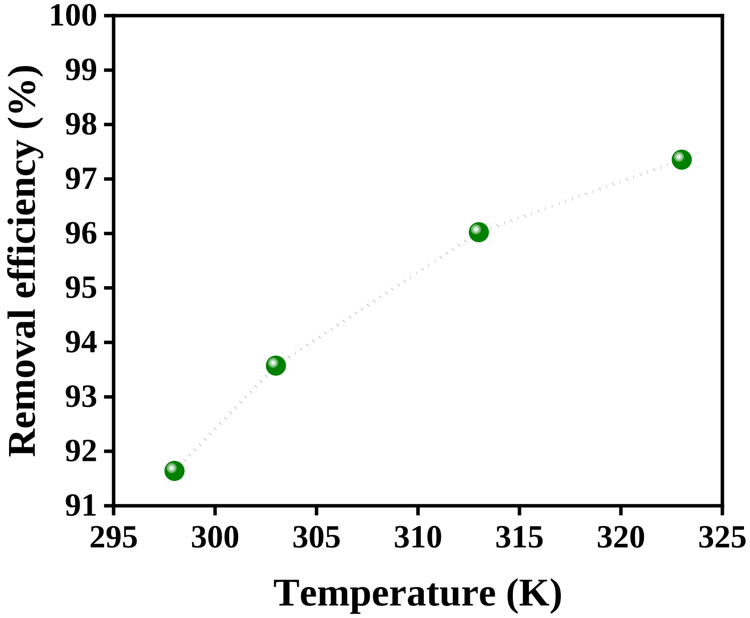

Figure S2. Effect of temperature on the removal efficiency of Pb(II) onto the RGO-Ag_2MoO_4 NCs.

References

1. Xiong, C.; Wang, S.; Zhang, L. Selective recovery mechanism of Au(III) from an aqueous solution by trimethyl phosphate modified poly(glycidyl methacrylate). *Journal of the Taiwan Institute of Chemical Engineers* **2019**, *95*, 55-64, doi:https://doi.org/10.1016/j.jtice.2018.09.035.
2. Xiong, C.; Wang, S.; Hu, P.; Huang, L.; Xue, C.; Yang, Z.; Zhou, X.; Wang, Y.; Ji, H. Efficient selective removal of Pb (II) by using 6-aminothiouracil-modified Zr-based organic frameworks: from experiments to mechanisms. *ACS applied materials & interfaces* **2020**, *12*, 7162-7178.
